# Clinical characteristics and online mental health care of asymptomatic or mildly symptomatic patients with coronavirus disease 2019

**DOI:** 10.1371/journal.pone.0242130

**Published:** 2020-11-23

**Authors:** Su Jin Jeong, Won Suk Chung, Yujin Sohn, Jong Hoon Hyun, Yae Jee Baek, Yunsuk Cho, Jung Ho Kim, Jin Young Ahn, Jun Yong Choi, Joon-Sup Yeom

**Affiliations:** 1 Department of Internal Medicine, Severance Hospital, Yonsei University College of Medicine, Seoul, South Korea; 2 Yonsei University Graduate School, Seoul, South Korea; University of São Paulo, BRAZIL

## Abstract

Comparing to data in patients with severe coronavirus diseases 2019 (COVID-19), there are few studies on the prevalence anxiety and/or depression in patients with asymptomatic or mildly symptomatic COVID-19. We investigated the clinical characteristics and the prevalence of anxiety and/or depression among asymptomatic or mildly symptomatic patients with COVID-19 and monitored their mental health using an online assessment. An online survey for monitoring and assessing the mental health of patients with COVID-19 using a mobile phone was conducted. We used the Hospital Anxiety and Depression Scale to measure anxiety and/or depression levels. Of the 234 patients, 66 patients were asymptomatic (28.2%), while the remaining 168 patients were mildly symptomatic. The prevalence of anosmia (*p* = 0.001) and ageusia (*p* = 0.008) significantly decreased with the increasing age. In addition, 19.8% and 14.0% patients had anxiety and/or depression in the first survey, and one week after the first survey, respectively. Compared to patients without anxiety and/or depression, those with anxiety and/or depression had a longer quarantine duration. We found that anomia and ageusia were relatively common in the young age group. Furthermore, one-fifth asymptomatic or mildly symptomatic patients with COVID-19 had anxiety and/or depression.

## Introduction

By the end of 2019, early cases of coronavirus disease 2019 (COVID-19) were first reported in Wuhan, China and COVID-19 had started spreading throughout China [1, 2]. The novel coronavirus causing of COVID-19 shares genomic sequence identity with both severe acute respiratory syndrome coronavirus (SARS-CoV) and Middle East respiratory syndrome coronavirus (MERS-CoV) [3]. In addition, the epidemiology of COVID-19 is similar to that of severe acute respiratory syndrome (SARS); hence, and the novel coronavirus has been named severe acute respiratory syndrome coronavirus 2 (SARS-CoV-2) [3, 4]. Despite undertaking strict quarantine measures, COVID-19 has spread rapidly worldwide. In March 2020, the World Health Organization has declared the COVID-19 outbreak a pandemic.

In South Korea, the surge of confirmed cases was recognized from February 2020. To allocate medical resources efficiently, the South Korean government classified patients according to the severity of COVID-19, and asymptomatic or mildly symptomatic patients were admitted to the community treatment centers (CTCs), non-hospital facilities for isolation and monitoring. COVID-19 can manifest either as an asymptomatic infection or mild-to-severe pneumonia. Data pertaining to the clinical characteristics and mental health support are limited in patients who asymptomatic or had mild symptoms, who constitute the base of the disease pyramid, comparing to data in patients with severe COVID-19 symptoms [5].

Therefore, we analyzed the clinical characteristics of asymptomatic or mildly symptomatic patients with COVID-19. We also monitoring their mental health using an online assessment during the COVID-19 outbreak.

## Methods

### Patients and study settings

We included patients admitted to the CTC with asymptomatic or mildly symptomatic COVID-19 based on the early warning score of <3 for SARS-CoV-2 infected patients [6]. In this study, patients referred to other hospitals due to a symptom change during the isolation were excluded.

A written informed consent was obtained from each patient who agreed to participate in the online mental health assessment. Missing data were collected using telephone interviews by well-trained doctors. This study was approved by the Institutional Review Board of Severance Hospital (Seoul, South Korea).

We conducted an online survey for the monitoring of patients with COVID-19 from March 15 to April 10, 2020. And, we also examined the mental health of the patients using an online assessment once a week for 2 weeks during the quarantine period.

### Definition

Reverse transcription polymerase chain reaction (RT-PCR) assays for the *E* (envelope protein), *RdRP* (RNA-dependent RNA polymerase), and *N* (nucleocapsid protein) genes were performed with the Allplex^™^ 2019-nCoV Assay (Seegene Inc., Seoul, South Korea) using nasopharyngeal swab samples. Negative RT-PCR results were re-tested by the RT-PCR after 24 hours; positive RT-PCR results were re-tested by RT-PCR after 7days; and inconclusive RT-PCR results were re-tested by RT-PCR after 3days.

Results of the RT-PCR assays were expressed as the cycle threshold (Ct) value. Negative RT-PCR results were defined as Ct values ≥40. Negative conversion was defined as two consecutive negative RT-PCR results with a 24-hour interval.

The patients were divided into 4 groups by age patients aged <20 years were categorized in group A; those aged 20–39 years were categorized in group B; those aged 40–59 years were categorized in group C; and those aged ≥60 years categorized in the group D.

The Hospital Anxiety and Depression Scale (HADS), a self-assessment scale using an online survey, was used to measure the levels of anxiety and depression [7]. Anxiety and depression were assessed respectively using the 7-item subscales, which were scored from 0 to 21 for each subscale. Scores of 11–21 indicate an abnormal case (probable case of anxiety or depression) [8]. HADS was administered after the second week of the quarantine period, and a follow-up survey was conducted one week after the first survey.

### Statistical analysis

All variables are expressed as the mean ± standard deviation (SD), unless otherwise indicated. Statistical significance was set at the level of *p* < 0.05. Categorical variables were compared using χ^2^ test, and continuous variables with normal distributions were compared using the Student’s *t*-test. Differences in the four age groups were evaluated by one-way ANOVA with Tukey’s multiple comparison test. All statistical analyses were performed using IBM SPSS Statistics for Windows, version 23.0 (IBM Corp., Armonk, NY, USA).

## Results

### Clinical characteristics

An analysis was conducted on 234 of 247 patients with COVID-19 excluding 13 patients who referred to other hospitals due to a symptom change during the isolation. The mean age of all patients was 37.78 ± 15.57 years, and 93 of the study participants were male (39.7%). Among them, there were 66 (28.2%) asymptomatic patients with COVID-19.

**Fig 1:**
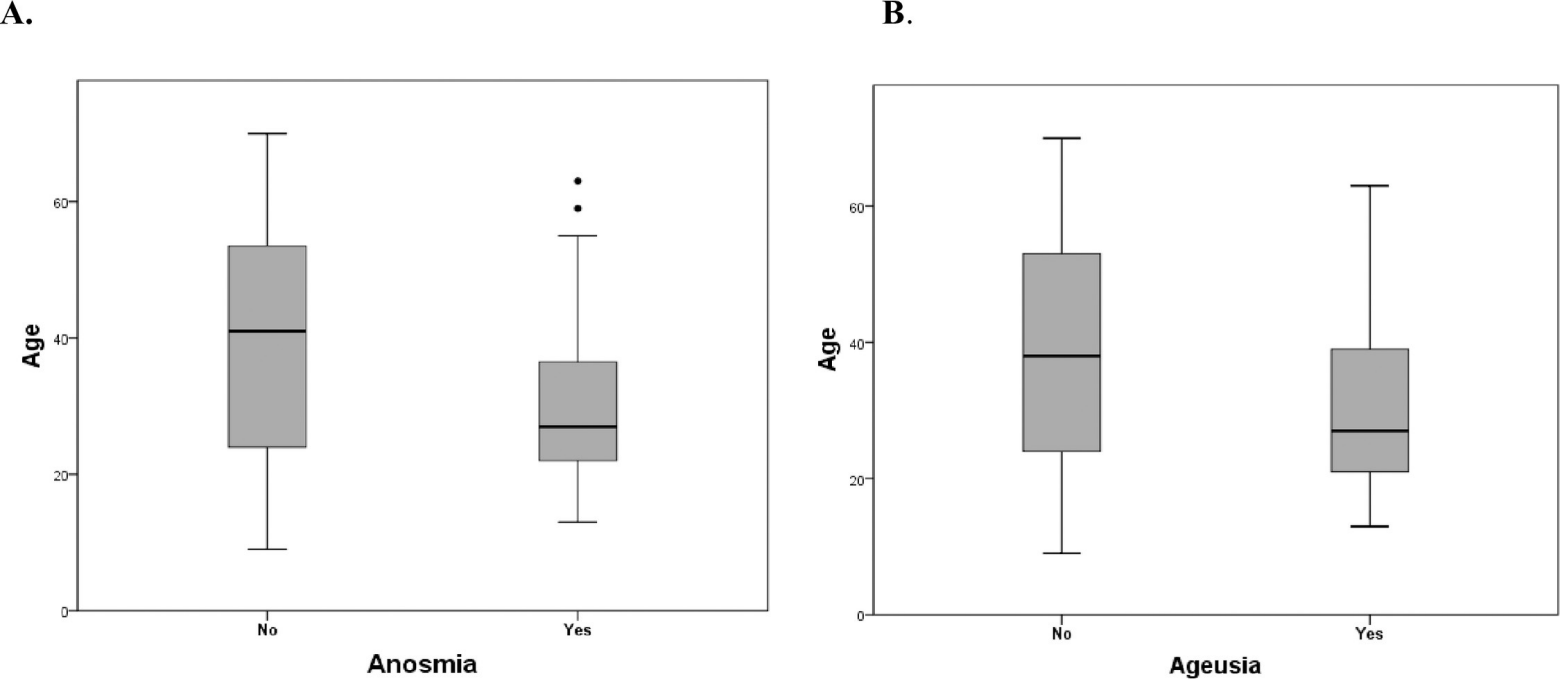
Distribution of age according to patients without or with symptoms. **A**. Patients with anosmia (n = 47), **B.** Patients with ageusia (n = 37).

Clinical characteristics of the patients stratified by age are summarized in Table 1. Fever was present in 17.5% of the patients. The most common symptoms were myalgia (23.9%), anosmia (20.1%), and cough (19.7%); chest pain (1.7%) and vomiting (1.7%) were uncommon. Anosmia (*p* = 0.001) and ageusia (*p* = 0.008) significantly decreased with increasing age. In addition, nasal stuffiness (*p* = 0.027), dizziness (*p* = 0.020), and headaches (*p* = 0.010) showed a statistically significant decrease in older age groups. Fig 1 shows the distribution of age according to symptom status (anosmia and ageusia). Patients with anosmia were younger than those without anosmia (mean age: 30.60 ± 12.55 vs. 39.59 ± 15.76 years, *p* < 0.001); patients with ageusia were younger than those without symptoms (mean age: 31.43 ± 14.22 vs. 38.97 ± 15.56 years, *p* = 0.005).

**Table 1:** Comparisons of demographic and clinical characteristics according to stratified age groups in asymptomatic or mildly symptomatic patients with COVID-19.

Variables	Total (n = 234)	Group A (n = 33)	Group B (n = 97)	Group C (n = 85)	Group D (n = 19)	*p* value
Age, years	37.78 ± 15.57	18.03 ± 2.53	27.48 ± 5.23	51.54 ± 5.03	63.11 ± 2.18	
Male, yes	93 (39.7)	15 (45.5)	49 (50.5)	23 (27.1)	6 (31.6)	0.011
Smoker	22 (9.4)	4 (12.1)	11(11.3)	5 (5.9)	2 (10.5)	0.349
Comorbidity						
Hypertension	15 (6.4)	0 (0.0)	5 (5.2)	8 (9.4)	2 (10.5)	0.044
Diabetes	2 (0.9)	0 (0.0)	0 (0.0)	1 (1.2)	1 (1.2)	0.055
COPD	3 (1.3)	0 (0.0)	0 (0.0)	2 (2.4)	1 (5.3)	0.046
Old tuberculosis	3 (1.3)	0 (0.0)	0 (0.0)	3 (3.5)	0 (0.0)	0.195
Asthma	3 (1.3)	0 (0.0)	2 (2.1)	1 (1.2)	0 (0.0)	0.914
Allergy	12 (5.1)	4 (12.1)	6 (6.2)	2 (2.4)	0 (0.0)	0.018
Chronic kidney disease	1 (0.4)	0 (0.0)	1 (1.0)	0 (0.0)	0 (0.0)	0.641
Chronic liver disease	3 (1.3)	0 (0.0)	0 (0.0)	3 (3.5)	0 (0.0)	0.195
Malignancy	7 (3.0)	0 (0.0)	0 (0.0)	5 (5.9)	2 (10.5)	0.003
Symptomatic	120 (51.3)	20 (60.6)	53 (54.6)	39 (45.9)	8 (42.1)	
Symptomatic, early relief*	48 (20.5)	3 (9.1)	20 (20.6)	22 (25.9)	3 (15.8)	
Asymptomatic	66 (28.2)	10 (30.3)	24 (24.7)	24 (28.1)	8 (42.1)	0.499
Fever	41 (17.5)	11 (33.3)	14 (14.4)	14 (16.5)	2 (10.5)	0.068
Chill	37 (15.8)	7 (21.2)	10 (10.3)	16 (19.3)	4 (22.2)	0.482
Cough	46 (19.7)	6 (18.2)	25 (25.8)	11(12.9)	4 (21.1)	0.351
Sputum	40 (17.1)	5 (15.2)	21 (21.6)	11 (12.9)	3 (15.8)	0.477
Sore throat	31 (13.2)	1 (3.0)	16 (16.5)	12 (14.1)	2 (10.5)	0.473
Rhinorrhea	33 (14.1)	3 (9.1)	21 (21.6)	7 (8.2)	2 (10.5)	0.287
Nasal stuffiness, [T**]	37 (15.8)	4 (12.1), [a, b]	26 (26.8), [a]	6 (7.1), [a, b]	1 (5.3), [b]	0.027
Dizziness	11 (4.7)	3 (9.1)	7 (7.2)	1 (1.2)	0 (0.0)	0.020
Headache	11 (4.7)	8 (24.2)	24 (24.7)	10 (11.8)	1 (5.3)	0.010
Anosmia, [T**]	47 (20.1)	10 (30.3), [a]	27 (27.8), [a]	9 (10.6), [a, b]	1 (5.3), [b]	0.001
Ageusia	37 (15.8)	9 (27.3)	19 (19.6)	7 (8.2)	2 (10.5)	0.008
Myalgia	56 (23.9)	6 (18.2)	15 (15.5)	29 (34.1)	6 (31.6)	0.013
Fatigue	30 (12.8)	6 (18.2)	15 (15.5)	7 (8.2)	2 (10.5)	0.122
Chest pain	4 (1.7)	2 (6.1)	2 (2.1)	0 (0.0)	0 (0.0)	0.031
Vomiting	4 (1.7)	2 (6.1)	0 (0.0)	2 (2.4)	0 (0.0)	0.348
Diarrhea	15 (6.4)	2 (6.1)	8 (8.2)	3 (3.5)	2 (10.5)	0.804

COPD, chronic obstructive lung disease

*Early relief was defined as patients who have lost symptoms within 7 days.

**The same letters indicate non-significant difference between the groups based on Tukey’s multiple comparison test.

Patients were divided into Group A, B, C, and D (aged <20, 20–39, 40–59, and ≥60 years, respectively)

Continuous variables are shown as the mean ± standard deviation (SD) and categorical variables, as numbers (percentage).

**Fig 2:**
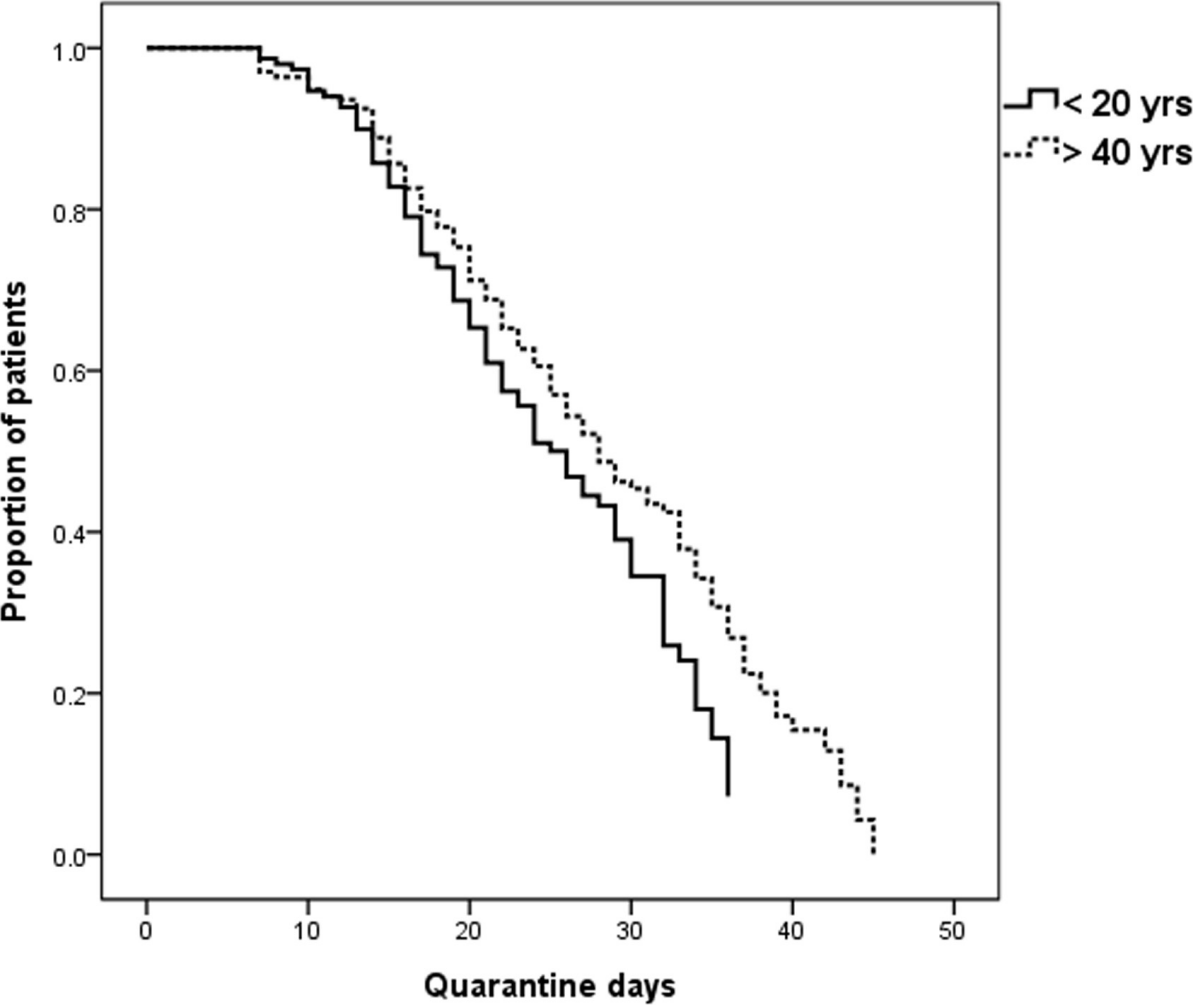
Quarantine time course of patients without negative conversion according to age (*p* = 0.014).

The quarantine time course of patients without negative conversion according to age is shown in Fig 2. The proportion of patients with negative conversion was significantly higher in the young age group (< 20 years) than in the old age group (>40 years) (*p* = 0.014).

### HADS survey for mental health care

Table 2 shows the results of the HADS survey for mental health care. Of the 234 patients, 146 patients (62.4%) participated and finished the questionnaires more than once; 66 patients (45.2%) completed the questionnaires twice and 80 patients (54.8%) participated in one of the two surveys. In the first survey, 25 of the 126 patients (19.8%) had anxiety and/or depression (13 cases of anxiety and 20 cases of depression). In the second survey, 12 of the 86 patients (14.0%) had anxiety and/or depression (3 cases of anxiety, and 11 cases of depression). Among patients who finished the questionnaires twice, the mean anxiety score in the second survey decreased compared to that in the first survey.

**Table 2:** Results of Hospital Anxiety and Depression Scale survey in asymptomatic or mildly symptomatic patients with COVID-19.

Variables	First survey (n = 126)	Second survey (n = 86)	*p* value
Age, years	36.2 ± 15.26	34.46 ± 15.2	0.403
Quarantine period, days	23.75 ± 5.16	30.56 ± 4.56	-
Anxiety score	5.38 ± 3.80	4.37 ± 3.13	0.036
Depression score	6.70 ± 4.02	6.10 ± 4.21	0.302
Abnormal cases*	25^a^/126 (19.8)	12^b^/86 (14.0)	0.267
Consultation with experts	3/126 (2.4)	1/86 (1.2)	0.648
Patients with both tests (n = 66)			
Age, years (36.5 ± 15.6)			
Quarantine period, days**	23.85 ± 4.64	30.85 ± 4.64	-
Anxiety score**	4.88 ± 3.18	4.21 ± 2.90	0.211
Depression score**	6.20 ± 3.93	6.20 ± 3.86	1.000

*A score of 11 or higher indicated probable presence of the mood disorder.

^a^13 cases in anxiety scores, and 20 cases in depression scores.

^b^3 cases in anxiety scores, and 11 cases in depression scores.

**It shows only the value of patients who conducted both surveys (n = 66).

Continuous variables are shown as the mean ± standard deviation (SD) and categorical variables, as numbers (percentage).

In the first survey, age and quarantine days were compared between patients with and without anxiety and/or depression. Compared to patients (101/126,80.2%) without anxiety and/or depression, those with anxiety and/or depression (25/126, 19.8%) had a longer duration of quarantine (25.52 ± 4.78 vs. 23.32 ± 5.17 days; *p* = 0.007). Patients with anxiety and/or depression were older (than those without anxiety and/or depression 38.28 ± 14.22 vs. 35.76 ± 15.76 years; *p* = 0.463), but the difference was not statistically significant. In the second survey, there were no significant differences in age and quarantine days between the two groups.

## Discussion

In the present study, we provided the demographic and clinical data of 234 asymptomatic or mildly symptomatic patients with COVID-19 who were only isolated at the CTC and were not administered any COVID-19 treatment. None of the 234 individuals developed pneumonia, and 66 patients (28.2%) were asymptomatic during the isolation.

The precise interval during which a patient with COVID-19 is infectious is uncertain. It appears that SARS-CoV-2 can be transmitted prior to the onset of symptoms and throughout the course of illness. Moreover, the transmission of SARS-CoV-2 from asymptomatic patients has also been reported [9, 10]. In addition, the highest viral RNA levels were detected from upper respiratory specimens soon after symptom onset compared; the levels subsequently declined over the course of the illness [11–13]. Therefore, to control this pandemic, rapid and large-scale RT-PCR testing needs to be conducted for identifying asymptomatic patients with COVID-19 through close contact tracing.

In our patients, the most common symptom was myalgia, followed by anosmia and cough, while fever and sputum production were observed in 17.5% and 17.1% patients, respectively. We found that mildly symptomatic patients in the young age group (<40 years) were prone to have anosmia and ageusia during isolation, and these symptoms were accompanied by nasal stuffiness, dizziness, or headaches. Particularly, smell and taste dysfunctions have been reported as uncommon symptoms of COVID-19 in early Chinese reports. In a previous study, the frequency of neurological manifestation was analyzed in 214 patients with COVID-19; anosmia and ageusia were noted in 11 (5.1%) and 12 (5.6%) patients, respectively [14]. Some studies conducted in Europe have detected a very high frequency (19.4%–88%) of chemosensitive disorders in patients with COVID-19 [15–18].

Anosmia has been reported in SARS [19] and other coronavirus infections [20]; however, it is a rare occurrence. Anosmia might be caused due to the direct damage of the olfactory and gustatory receptors caused by the virus [21]. In our study, even mildly symptomatic patients had anosmia or ageusia in the early stage of COVID-19, and patients with anosmia were younger than those without anosmia. In other studies, the olfactory and gustative alterations were reported more frequently in the early stages of the infection and in mildly symptomatic patients [15–17], which is consistent with our study results. Therefore, anosmia and ageusia could represent crucial symptoms for suspecting SARS-COV-2 infection.

In our study, the prevalence of anxiety and/or depression was 19.8% among asymptomatic or mildly symptomatic patients with COVID-19. Only limited studies have reported mental health care of asymptomatic or mildly symptomatic patients with COVID-19. The negative impact of emerging infectious diseases has resulted in mental health sequelae, including distress, insomnia, anxiety, depression, and posttraumatic stress disorder [22]. A recent meta-analysis and empirical analysis of psychological burden scales to assess the impact of isolation precautions on the quality of life showed that the effect in all studies, except one, was negative and that isolated patients are the worst affected [23].

We found that patient who completed the survey twice exhibited decreased anxiety scores, but it was not statistically significant. After the first and second surveys, general physicians from the CTC consulted patients with anxiety and/or depression over the phone; there was a decrease in the patients’ psychological distress. In total, 4 patients wanted to consult a psychiatrist (3 patients from the first survey and 1 patient from the second survey). One of 4 patients who wanted expert counseling took a medicine (anxiolytics), and the others improved only through on-line supportive psychotherapy.

In the early stages of the SARS outbreak, a range of psychiatric conditions, including high levels of stress, depressive symptoms, anxiety, insomnia, nightmares, and poor concentration were reported by SARS patients [24]. These psychiatric morbidities might be still significant even after the physical recovery of SARS-infected individuals [25]. Psychosocial consequences have also been reported in COVID-19 patients [26].

Furthermore, Patients who had recovered from SARS and a history of psychiatric consultations in the acute phase of illness had a higher risk for psychological distress later [22]. Therefore, patients with SARS-CoV-2 infection should be provided timely and focused mental health support after their discharge from quarantine. On the other hand, the mental health may be serious not just for the patients but also for others who are in the general population, among health care workers, and in vulnerable population [27, 28]. The COVID-19 pandemic has led to a vigorous and multifaceted response from psychiatrists and other professionals. The effect of pandemic on mental health will remain for a long time after COVID-19 outbreaks. Therefore, there is a need for in-depth research and guidelines of management in the general or vulnerable population [27].

who are already vulnerable to biological or psychosocial stressors, health care workers, and even people who are following the news through numerous media channels in pandemics.

This study has some limitations. First, it is limited by its small sample size and using simple scale for mental health check. The COVID-19 outbreak is leading to widespread fear, anxiety, and other psychological distress. Therefore, it is necessary to apply various psychometric assessment for an in-depth analysis. Further large-scale and in-depth studies of mental health care in a diverse clinical spectrum of patients with SARS-CoV-2 infection are required. Second, the Ct values were not collected at the time of diagnosis. Therefore, we could not analyze the serial changes in Ct values for the three genes in asymptomatic or mildly symptomatic patients with COVID-19. Third, this study did not include the clinical outcomes of all the patients because some of the patients were referred to another CTC at the end of the quarantine period; hence, further analysis would be needed. Fourth, we collected the mental health data of patients using a mobile phone-based survey, and, for patients who could not use their mobile phones, physicians asked direct questions by telephone. Therefore, there might be a data discrepancy between the two groups.

This is the first study to describe the mental health status in asymptomatic or mildly symptomatic patients with COVID-19 in South Korea. We found that 66 (28.2%) patients were asymptomatic and that anomia and ageusia were relatively common in the young age group. The demographic and clinical characteristics of asymptomatic or mildly symptomatic patients with COVID-19, including the mental health status presented in this study can contribute in developing guidelines for controlling COVID-19.

## Supporting information

S1 File (PDF)

S2 File (DOCX)

S3 File (XLSX)
