# Supplementary material for: Clinical characteristics and online mental health care of asymptomatic or mildly symptomatic patients with coronavirus disease 2019
Source: PLoS One. 2020 Nov 23;15(11):e0242130. doi: 10.1371/journal.pone.0242130 (PMC7682865; doi:10.1371/journal.pone.0242130)
Supplement: S1 File — (PDF) [file pone.0242130.s001.pdf]

Hospital Anxiety and Depression Scale (HADS)

Tick the box beside the reply that is closest to how you have been feeling in the past week.
Don't take too long over you replies: your immediate is best.

D	A	
		**I feel tense or 'wound up':**
	3	Most of the time
	2	A lot of the time
	1	From time to time, occasionally
	0	Not at all
		**I still enjoy the things I used to enjoy:**
0		Definitely as much
1		Not quite so much
2		Only a little
3		Hardly at all
		**I get a sort of frightened feeling as if something awful is about to happen:**
	3	Very definitely and quite badly
	2	Yes, but not too badly
	1	A little, but it doesn't worry me
	0	Not at all
		**I can laugh and see the funny side of things:**
0		As much as I always could
1		Not quite so much now
2		Definitely not so much now
3		Not at all
		**Worrying thoughts go through my mind:**
	3	A great deal of the time
	2	A lot of the time
	1	From time to time, but not too often
	0	Only occasionally
		**I feel cheerful:**
3		Not at all
2		Not often
1		Sometimes
0		Most of the time
		**I can sit at ease and feel relaxed:**
	0	Definitely
	1	Usually
	2	Not Often
	3	Not at all

D	A	
		**I feel as if I am slowed down:**
3		Nearly all the time
2		Very often
1		Sometimes
0		Not at all
		**I get a sort of frightened feeling like 'butterflies' in the stomach:**
	0	Not at all
	1	Occasionally
	2	Quite Often
	3	Very Often
		**I have lost interest in my appearance:**
3		Definitely
2		I don't take as much care as I should
1		I may not take quite as much care
0		I take just as much care as ever
		**I feel restless as I have to be on the move:**
	3	Very much indeed
	2	Quite a lot
	1	Not very much
	0	Not at all
		**I look forward with enjoyment to things:**
0		As much as I ever did
1		Rather less than I used to
2		Definitely less than I used to
3		Hardly at all
		**I get sudden feelings of panic:**
	3	Very often indeed
	2	Quite often
	1	Not very often
	0	Not at all
		**I can enjoy a good book or radio or TV program:**
0		Often
1		Sometimes
2		Not often
3		Very seldom

Please check you have answered all the questions

Scoring:

Total score: Depression (D) ___________ Anxiety (A) ______________

0-7 = Normal

8-10 = Borderline abnormal (borderline case)

11-21 = Abnormal (case)
