# Supplementary material for: Clinical characteristics and online mental health care of asymptomatic or mildly symptomatic patients with coronavirus disease 2019
Source: PLoS One. 2020 Nov 23;15(11):e0242130. doi: 10.1371/journal.pone.0242130 (PMC7682865; doi:10.1371/journal.pone.0242130)
Supplement: S2 File — (DOCX) [file pone.0242130.s002.docx]

| **당신의 상태를 가장 잘 나타낸다고 생각되는 문항에**  **[●] 체크해 주세요.** | |
| --- | --- |
| **나는 긴장감 또는 “정신적 고통”을 느낀다.** | **나는 즐겨오던 것들을 현재도 즐기고 있다.** |
| **ⓞ 전혀 아니다.**  **① 가끔 그렇다.**  **② 자주 그렇다.**  **③ 거의 그렇다.** | **ⓞ 똑같이 즐긴다.**  **① 많이 즐기지는 못한다.**  **② 단지 조금만 즐긴다.**  **③ 거의 즐기지 못한다.** |
| **무언가 무서운 일이 일어날 것 같은 느낌이 든다.** | **나는 사물을 긍정적으로 보고 잘 웃는다.** |
| **ⓞ** 전혀 아니다.  **①** 조금 있지만 걱정하지 않는다.  **②** 있지만 그렇게 나쁘지는 않다.  **③** 매우 분명하고 기분이 나쁘다. | **ⓞ** 나는 항상 그렇다.  **①** 현재는 그다지 그렇지 않다.  **②** 거의 그렇지 않다.  **③** 전혀 아니다. |
| **마음 속에 걱정스러운 생각이 든다.** | **나는 기분이 좋다.** |
| **ⓞ** 거의 그렇지 않다.  **①** 가끔 그렇다.  **②** 자주 그렇다.  **③** 항상 그렇다. | **ⓞ** 항상 그렇다.  **①** 자주 그렇다.  **②** 가끔 그렇다.  **③** 전혀 그렇지 않다. |
| **나는 편하게 긴장을 풀 수 있다.** | **나는 기력이 떨어진 것 같다.** |
| **ⓞ** 항상 그렇다.  **①** 대부분 그렇다.  **②** 대부분 그렇지 않다.  **③** 전혀 그렇지 않다. | **ⓞ** 전혀 아니다.  **①** 가끔 그렇다.  **②** 자주 그렇다.  **③** 거의 항상 그렇다. |
| **나는 초조하고 두렵다.** | **나는 나의 외모에 관심을 잃었다.** |
| **ⓞ** 전혀 아니다.  **①** 가끔 그렇다.  **②** 자주 그렇다.  **③** 매우 자주 그렇다. | **ⓞ** 여전히 관심이 있다.  **①** 전과 같지는 않다.  **②** 이전보다 확실히 관심이 적다.  **③** 확실히 잃었다. |
| **나는 가만히 있지 못하고 안절부절 못한다.** | **나는 일들을 즐거운 마음으로 기대한다.** |
| **ⓞ** 전혀 아니다.  **①** 가끔 그렇다.  **②** 자주 그렇다.  **③** 매우 그렇다. | **ⓞ** 내가 전에 그랬던 것처럼 그렇다.  **①** 전보다 조금 덜 그렇다.  **②** 전보다 확실히 덜 그렇다.  **③** 전혀 그렇지 않다. |
| **나는 갑자기 당황스럽고 두려움을 느낀다.** | **나는 좋은 책, 라디오, 텔레비전을 즐길 수 있다.** |
| **ⓞ** 전혀 그렇지 않다.  **①** 가끔 그렇다.  **②** 꽤 자주 그렇다. **③** 거의 항상 그렇다. | **ⓞ** 자주 즐긴다.  **①** 가끔 즐긴다.  **②** 거의 못 즐긴다.  **③** 전혀 못 즐긴다. |

| **불안 점수 [ ]** | **우울 점수 [ ]** |
| --- | --- |

| 불안 점수와 우울 점수가 각각 **8점 이상**일 경우 불안과 우울에 대해 상담해 볼 것을 권유 드립니다. |
| --- |
